# Hospital Reconstruction and Rebuilding: Cost per Bed for Building Alone

**Published:** 1919-08-23

**Authors:** 


*To the Editor of* The Hospital.

### Hospital Reconstruction and Rebuilding: Cost per Bed for Building Alone.

*To the Editor of* The Hospital.

Sir,—I am on a committee that is considering hospital reconstruction and re-building, and thought perhaps you or your readers could give information as to the cost per bed of a new general hospital for building alone. Also are there any estimates of beds per thousand of population later than those of the Fabian Society and Dr. Fleming, of Bradford-on-Avon? We are beginning to recognise that there will have to be more beds in all the special departments than was thought likely only two years ago. Again, what part will Poor-Law infirmaries take in the general scheme?—I am, yours faithfully,

August 19, 1919. Surgeon.

